# On-Demand Telemedicine as a Disruptive Health Technology: Qualitative Study Exploring Emerging Business Models and Strategies Among Early Adopter Organizations in the United States

**DOI:** 10.2196/14304

**Published:** 2019-11-15

**Authors:** Ryan Sterling, Cynthia LeRouge

**Affiliations:** 1 Department of Health Services University of Washington Seattle, WA United States; 2 Department of Information Systems & Business Analytics College of Business Florida International University Miami, FL United States

**Keywords:** telemedicine, disruptive technology, business model, business strategy

## Abstract

**Background:**

On-demand telemedicine is increasingly adopted by health organizations to meet patient demand for convenient, accessible, and affordable services. Little guidance is currently available to new entrant organizations as they consider viable business models and strategies to harness the disruptive potential of on-demand telemedicine services (in particular, virtual urgent care clinics [VCCs] as a predominant and catalyst form of on-demand telemedicine).

**Objective:**

We recognized on-demand telemedicine as a disruptive technology to explore the experiences of early adopter organizations as they launch on-demand telemedicine services and deploy business models and strategies. Focusing on VCC service lines, this study addressed the following research questions: (1) what is the emerging business model being deployed for on-demand telemedicine?; (2) what are the core components of the emerging business model for on-demand telemedicine?; and (3) what are the disruptive business strategies employed by early adopter organizations as they launch on-demand telemedicine services?

**Methods:**

This qualitative study gathered data from 32 semistructured phone interviews with key informants from 19 VCC early adopter organizations across the United States. Interview protocols were developed based on noted dissemination and implementation science frameworks. We used the constant comparison method to transform study data into stable dimensions that revealed emerging business models, core business model components (value proposition, key resources, key processes, and profit formula), and accompanying business strategies.

**Results:**

Early adopters are deploying business models that most closely align with a value-adding process model archetype. By and large, we found that this general model appropriately matches resources, processes, and profit formulas to support the disruptive potential of on-demand telemedicine. In total, 4 business strategy areas were discovered to particularly contribute to business model success for on-demand disruption among early adopters: fundamental disruptions to the model of care delivery; outsourcing support for on-demand services; disruptive market strategies to target potential users; and new and unexpected organizational partnerships to increase return on investment.

**Conclusions:**

On-demand telemedicine is a potentially disruptive innovation currently in the early adopter stage of technology adoption and diffusion. On-demand telemedicine must cross into the early majority stage to truly be a positive disruption that will increase accessibility and affordability for health care consumers. Our findings provide guidance for adopter organizations as they seek to deploy viable business models and successful strategies to smooth the transition to early majority status. We present important insights for both early adopters and potential early majority organizations to better harness the disruptive potential of on-demand telemedicine.

## Introduction

### Background

Health care organizations in the United States are operating in a time of high volatility [1-6]. Contributing to current pressures is the rise of consumerism in health care, driving patient demand for convenient, accessible, and affordable services. To compete and thrive, many organizations are adopting telemedicine solutions [7]. Telemedicine involves the use of medical information exchanged from one site to another via electronic communications to improve a patient’s clinical health status [8]. In 2018, more than 50% of hospitals and health systems reported some form of telemedicine offering [7,9].

Whether telemedicine should be considered a disruptive technology is a topic of debate. Disruptive technologies are innovations that disrupt and displace established market leaders by offering products and services that are cheaper, simpler, and more convenient than what is currently available [10]. Those that assert telemedicine as a disruptive technology view it as a disruptive model of care delivery that challenges the status quo (ie, facility-based, in-person services) to create greater access and affordability in health care [11]; those in opposition view it as an innovation that improves, but ultimately sustains the performance trajectory of traditional market leaders in care delivery [12]. A holistic view of telemedicine as one health care service fuels the debate. In practice, telemedicine is not one health service offering, but actually a cadre of potential service lines, each with its own nuances in goals, workflow, stakeholders, and financing—much like in-person care.

Both practice and research may benefit from taking a closer look at forms of telemedicine that stand out as strong disrupter entrants if we want to successfully harness and leverage the potential of these service lines. It is our position that, in particular, some newer forms of telemedicine create a compelling case that they will disrupt current delivery models of medical care by offering a less-expensive, highly accessible, and more convenient alternative to many in-person options. Newcomer service lines often include offerings for *on-demand* telemedicine that are initiated by health care consumers [13]. In comparison with traditional modes of facility-based in-person care delivery, on-demand services are patient-initiated and accessible around-the-clock from any location [13]. These potential advantages may attract health organizations operating in high volatility environments, seeking ways to manage existing pressures, including the rise of consumerism. Indeed, recent research of telemedicine adoption rates and drivers indicates strong and growing interest for on-demand services that allow patients at home or on the go to reach a clinical provider for a nonemergency consult at a transparent and low-cost fee (typically US $30-50) [7].

Riding a tide of increased market growth and an uptick in adoption rates among health organizations, on-demand telemedicine may hold great promise as a disruptive technology that will bring greater accessibility and affordability to health care. However, little guidance is currently available to new entrants as they consider viable business models and strategies for on-demand services. On-demand telemedicine is in the early adopter stage of technology adoption and diffusion, with the potential trajectory of approaching early majority in the coming years [14]. This can be a precarious position for widespread assimilation of on-demand services, as the inability to bridge the innovation chasm between these stages is known to impact the success of disruptive technologies [14-16]. In general, for a disruptive technology to successfully cross into widespread assimilation, adopter organizations must understand how to navigate viable business models and strategies to expand market potential and encourage adoption among more cautious pragmatists [15,16]. Therefore, now is an opportune time to discover lessons learned from the experiences of early adopter organizations of on-demand telemedicine that are in the process of navigating these rocky waters. Few research studies in the telemedicine or disruptive technology domains provide strategy and practical guidance for those embarking on new telemedicine service lines [17,18]. Moreover, existing studies do not speak through the lens of disruptive technology to yield lessons from early adopters or detail specific forms of telemedicine [17,18].

**Figure 1:**
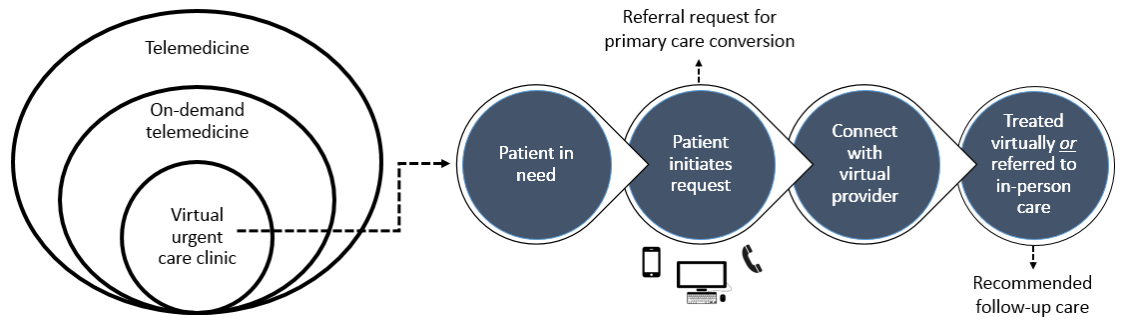
Virtual urgent care clinic encounter process.

There are many different forms of on-demand telemedicine, such as for primary care, behavioral health care, and urgent care. The virtual urgent care clinic (VCC) is a widely adopted form of the on-demand service that has received growing attention in the peer-reviewed literature [19-23]. Owing to this distinction, our study views VCC as a catalyst form of disruptive technology that can be used to examine on-demand service launch and business model deployment. VCC provides primary and urgent care services for nonemergent medical conditions that can be managed effectively by telemedicine, such as chronic bronchitis, conjunctivitis, rashes, and upper respiratory tract infections [13]. Figure 1 displays where VCC is situated in the wider context of telemedicine and reviews the general patient encounter process (see Multimedia Appendix 1 for additional information regarding the encounter process).

### Disruptive Technology Business Model

Although disruptive technologies have brought greater accessibility and affordability to consumers in other industries, the same cannot be widely said for the health care delivery sector [24-26]. Prior health care research suggests this failure is associated with misalignment between disruptive technologies and the need for business model innovation [25]. According to Hwang and Christensen [25]:

Legacy institutions of health care delivery are jumbled mixtures of multiple business models struggling to deliver value out of chaos…The health care system has trapped many disruption-enabling technologies in high-cost institutions that have conflated two and often three business models under the same roof. The situation screams for business model innovation.

It is well documented that the success of a disruptive technology is closely tied to its business model [27-32]. The business model provides a framework for an organization to create and capture value out of the disruption [27-29]. According to Johnson et al [27], pairing disruptive technologies with the right innovative business model can lead to greater accessibility and affordability. Research indicates that business models can be generally categorized into 3 archetypes: solution shops, value-adding processes, and facilitated user networks [25,26]. Table 1 provides an overview of these leading archetypes.

To better avoid the failures encountered by other disruptive technologies in the health care delivery sector, new information is needed regarding if and where on-demand telemedicine fits into the general topology of leading business model archetypes. The current landscape of experiences among early adopter health organizations can provide us insight into emerging business models. This leads us to our first research question: *what is the emerging business model being deployed for on-demand telemedicine (specifically, in the form of VCC)?*

**Table 1:** Overview of leading business model archetypes.

Characteristics	Business model archetypes		
	Solution Shop	Value-adding process	Facilitated user network
General model description	Used to diagnose and solve unstructured problems that are unique case to case. Value is derived from employees who diagnose causes and recommend solutions.	Used to transform inputs into outputs of greater value. Value is derived by using standardized inputs and uniform, convenient processes to produce consistent results.	Used for enterprises in which people exchange things with one another. Value is derived by facilitating the effective operation of the network.
Examples of model deployment	Consulting firms Advertising agencies Diagnostic work performed in general hospitals	Automobile manufacturing Common medical procedures after definitive diagnosis	Mutual insurance companies eBay Behavioral health support groups

### Disruptive Technology Business Model Components

**Figure 2:**
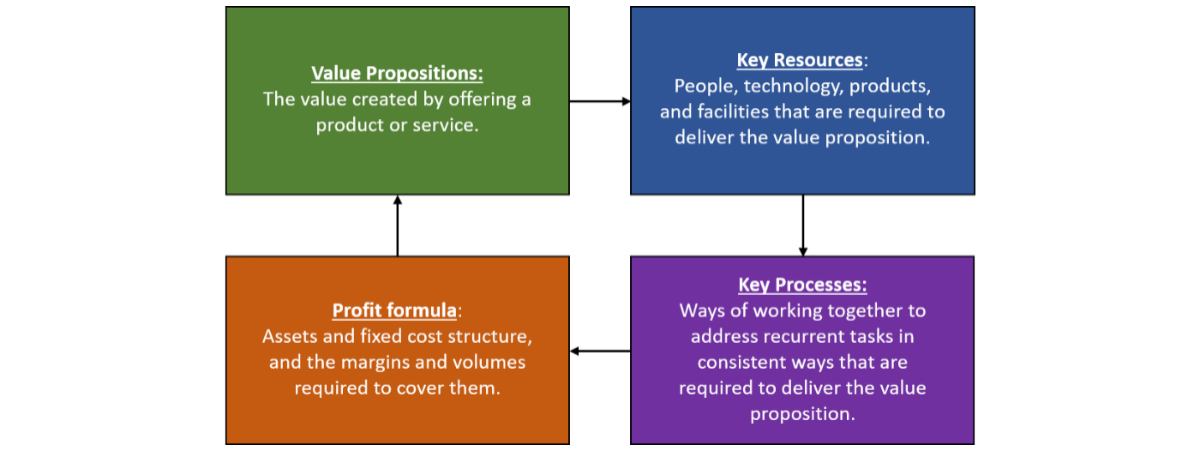
Business model framework components.

While useful, identifying a befitting type of business model does not provide the detail needed to inform strategic direction. Being leaders in the field, Johnson et al [27] understand any given business model as consisting of 4 interrelated strategic components (see Figure 2), including (1) the value proposition, or value created by offering a product or service, (2) key resources and (3) key processes that are needed to deliver the value proposition, and (4) the profit formula that defines how money is made for a deploying organization via delivery.

Once the 4 components coalesce into an established business model, only value propositions that fit the existing resources, processes, and profit formula can be successfully delivered [25]. Past disruptive technology research suggests these pieces must be fit together such that they are appropriately linked to an emerging disruptor for the new technology to succeed when brought to market [25]. More information is needed to specify these core components and their linkages in the context of on-demand telemedicine. To address this research gap, we propose our second research question: *what are the core components (value proposition, key resources, key processes, and profit formula) of the emerging business model for on-demand telemedicine (specifically, in the form of VCC)?*

### Disruptive Technology Business Strategies

While the business model describes the basic means by which an organization creates and delivers value from a disruptive technology, the business strategy is the specific method a deploying organization uses to achieve the proposed value and deal with opportunities and threats posed to the business model [28]. In the technology and innovation management field, little attention has been paid to the role of business strategies in association with emerging business models for disruptive technologies. More information is needed regarding what these disruptive strategies are and how they impact the path early adopters are taking to harness the potential of on-demand telemedicine. This leads us to our third research question: *what are the disruptive business strategies employed by early adopter organizations as they launch on-demand telemedicine services (specifically, in the form of VCC)*?

### Study Objective

**Figure 3:**
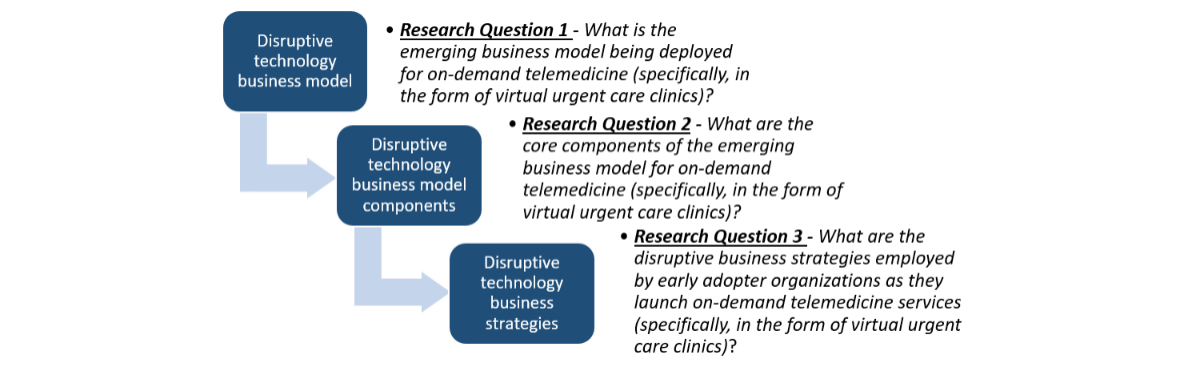
General research framework and specific research questions.

The objective of this qualitative study was to explore the paths that early adopters are taking to harness the disruptive potential of on-demand telemedicine, using VCC as a dominant instantiation. In doing so, we hoped to contribute to disruptive technology research by examining emerging business models and strategies being coupled with on-demand telemedicine services. We also aimed to offer practical guidance for adopter organizations as they seek to overcome some of today’s leading health care challenges using disruptive telemedicine solutions. Our general research framework and specific research questions are shown in Figure 3. To our knowledge, the components of this framework have never before been studied either collectively or independently in the context of on-demand telemedicine.

## Methods

### Study Population and Data Sources

This qualitative study focuses on a study population of VCC early adopter organizations nationwide. Participants represent a range of organizational types and geographic service areas from across the United States. Table 2 provides descriptive information regarding participant organizations. In total, 5 vendor organizations are represented in our study sample (including many leading vendors among the limited number of companies currently operating in the VCC market). Among nonvendor participants, most of the organizations have contracts with vendors to provide some degree of clinical staffing and technology infrastructure to support their VCC programs.

After a 6-month national recruitment effort, we developed points of contact at 25 organizations that offer VCC services; of that total, 19 organizations (19/25, 76%) agreed to participate in our study. Convenience and purposive sampling were used to identify potential VCC adopter organizations. We targeted potential participant organizations using contact lists from the American Telemedicine Association and National Consortium of Telehealth Resource Centers. We also used Web searches to identify other organizations that may not have been listed (using keyword searches for *telemedicine*, *telehealth*, *virtual clinic*, and other related terms); Web searches resulted in identification of 2 additional participant organizations. Overall, early adopter organizations stated they were eager to participate in the confidential interview process; organizations were interested in learning from our collective, deidentified findings in publication as a means of further advancing their VCC program efforts. Among the 6 organizations that declined to participate, most of them declined because of scheduling constraints among potential key informants.

Data sources included one-hour semistructured phone interviews with key informants from participating organizations and their organization’s VCC-related Web and print content. As staffing titles varied across participating organizations, organizational contacts assisted us in identifying key informants for study interviews. To recruit key informants, we targeted organizational roles related to strategy/business development, implementation, marketing, administrative operations, and clinical operations.

In total, 2 members of the research team conducted 32 phone interviews from September 2017 to December 2018. To promote an open and candid discussion, verbal and written recruiting messages emphasized confidentiality and the ability of the participants to skip questions and to go *off-the-record* with certain comments. Furthermore, at the beginning of each interview, key informants were made aware that all information collected during the interview would be completely confidential: anyone that was referred to during the interview would not be mentioned by name, nor would organizations be identified by name. All interviews were recorded (upon permission from key informants), deidentified, and transcribed before analysis. If there were any comments key informants did not wish to have recorded, the interview was postponed until all recording functions were turned off (*off-the-record*). Conversations were fluid, with few *off-the-record* requests. Failure to respond to a question was typically because of perceived lack of knowledge or factual detail related to the question; in most cases, a follow-up communication (eg, email) provided a response or a referral was made to a knowing person.

To provide breadth and depth of coverage, interview protocols were developed based on noted dissemination and implementation science frameworks that have been widely used to study the adoption of technologies in service delivery organizations, namely Damschroder Consolidated Framework for Implementation Science Research [33], Greenhalgh’s framework for diffusion of innovations in service organizations [34], and Aaron’s conceptual model of evidence-based practice implementation in public service sectors [35]. Collectively, these frameworks reflect a broad, sociotechnical organizational perspective that shaped our interview questions and allowed for an evidence-based exploration of business model and strategy components. Before use among key informants, experienced qualitative researchers familiar with the health information technology field and health care administrators and clinicians with a connection to telemedicine duties, such as telemedicine directors and virtual providers, reviewed the interview protocol. Minor refinements were made to the protocol as a result of this expert review (see Multimedia Appendix 2 to review our general study protocol; this general protocol was adjusted as needed to tailor interview questions and perspective to the type of organization and role of key informant).

**Table 2:** Characteristics of the participating virtual urgent care clinics early adopter organizations.

VCC^a^ service characteristics		VCC early adopter organization type (n)			
		Health systems (n=12)	Primary care practice (n=1)	Insurer (n=1)	Vendor (n=5)
**US geographic coverage**					
	West	4	0	1	0
	Midwest	4	0	0	0
	East	4	1	0	0
	National	0	0	0	5
**Rural/urban service area**					
	Urban	0	0	0	0
	Rural	1	0	0	0
	Urban/rural	11	1	1	5
**Available VCC modalities**					
	Only real-time text	0	0	0	2
	Only real-time phone	0	1	0	0
	Only real-time video	3	0	0	1
	Real-time phone and video	9	0	1	2
**Vendor engagement (among nonvendors)**					
	Clinical staffing and other support services	10	0	1	N/A^b^
	Nonclinical staffing support	2	0	0	N/A
	No vendor engagement	0	1	0	N/A

^a^VCC: virtual urgent clinic care.

^b^N/A: not applicable.

### Analytic Approach

We used the constant comparison method to analyze qualitative data [36,37]. Interview transcripts and supplementary Web and print content were coded independently by 1 or more research team members. Our team first deductively used noted dissemination and implementation science frameworks to develop an a priori coding schema [33-35]. Researchers met regularly during this process to iteratively discuss initial coding and refine coding categories [38]. Intercoder disagreements were resolved by consensus resolution, using an external qualitative expert to act as an auditor who makes final determinations as needed. We then carried out axial coding to inductively collapse initial coding categories into aggregate, stable dimensions that revealed emerging business models, strategic components, and accompanying business strategies [38]. Embedded in our interviewing and coding procedures, validity and reliability of study data and interpretation were assessed following Lincoln and Guba criteria for evaluating interpretive research [39,40]. Reporting of qualitative data was guided by the Consolidated Criteria for Reporting Qualitative Research [41]. We used Dedoose software for all qualitative data management and analysis [42].

## Results

### Overview

Our analysis revealed an emerging business model among VCC early adopters that closely aligns with the value-adding process archetype introduced in Table 1. We will first share our findings regarding the general characteristics of this emerging model and detail its core strategic components. We then describe 4 business strategies revealed from our data that are particularly indicative of the disruptive potential of VCC services.

### The Emerging Business Model Deployed by Early Adopters

Identification of VCC as a value-adding process business model archetype was supported in a number of ways. First, interviewees described a general business model focused on delivering a consistent, high quality patient care experience that is quick, convenient, and highly accessibility. According to an interviewee regarding convenience, accessibility, and expediency:

First and foremost with [VCC], it’s all about the convenience of being able to do it over your phone, your mobile phone, and on-demand. And so I've got a problem…I've got pink eye, I need to get that taken care of, I can open up my mobile phone, open up my app and I can be seen you know in less than 10 minutes.

Regarding emphasis on consistent high-quality patient care, another interviewee commented:

We have defined protocols that we create based on the best literature and research out there on the appropriate way to treat patients [virtually]. We've also undertaken to hire very experienced clinicians.

Second, indicative of the value-adding process archetype, organizations described a rule-based and uniform encounter process initiated after a VCC provider makes a definitive clinical diagnosis. Finally, with few exceptions, interviewees reported having deployed a business model dependent on service volumes to generate profit derived from the VCC encounter process. Service volumes were attributable to the VCC encounter itself and downstream from recommended follow-up care or referrals resulting from the on-demand visit:

So the key indicators are numbers of visits, and that includes number of visits to the website, the number of people who start the process, number of people who complete a virtual clinic visit…and then we track people who are appointed with a new primary care doctor in our system… we look at the financial return on visits that we are tracking.

To generate volume, organizations often relied on direct-to-consumer marketing to potential users to raise awareness and drive service uptake. To accommodate the needs associated with increased service volumes, most of our participating early adopter organizations relied to some extent on vendor outsourcing to support key resource inputs for the on-demand service, such as VCC clinical staffing and/or technology infrastructure (see Table 2).

Interestingly, the collective experiences of our interviewees suggest that many early adopters are leveraging their initial investment in VCC services to explore new potential innovations in the on-demand telemedicine space that are using different business model type structures. These newly spawned innovations share elements commonly associated with the user-facilitated network business model archetype reviewed in Table 1, such as the exchange of communications and data between users, and profit generation via membership or user fees. For example, some participating organizations are cultivating VCC and other on-demand telemedicine patient user networks and technologies to manage the care of many chronic diseases. To illustrate, an interviewee described a diabetes self-management program that uses a phone-based text messaging platform to share and discuss disease management information with a wide patient community in real-time.

In addition, in alignment with the facilitated user network archetype, other participating organizations described emerging strategies to expand their membership-based service operations to increase profit generation. In such arrangements, early adopters contract with outside self-insured entities to offer VCC or other on-demand telemedicine services directly. For the self-insured entity, financial returns are achieved via improved employee health, lower employee absenteeism, and greater employee retention. For the on-demand service provider, financial returns emerge by building a larger client base.

### Core Strategic Components of the Emerging Business Model

**Figure 4:**
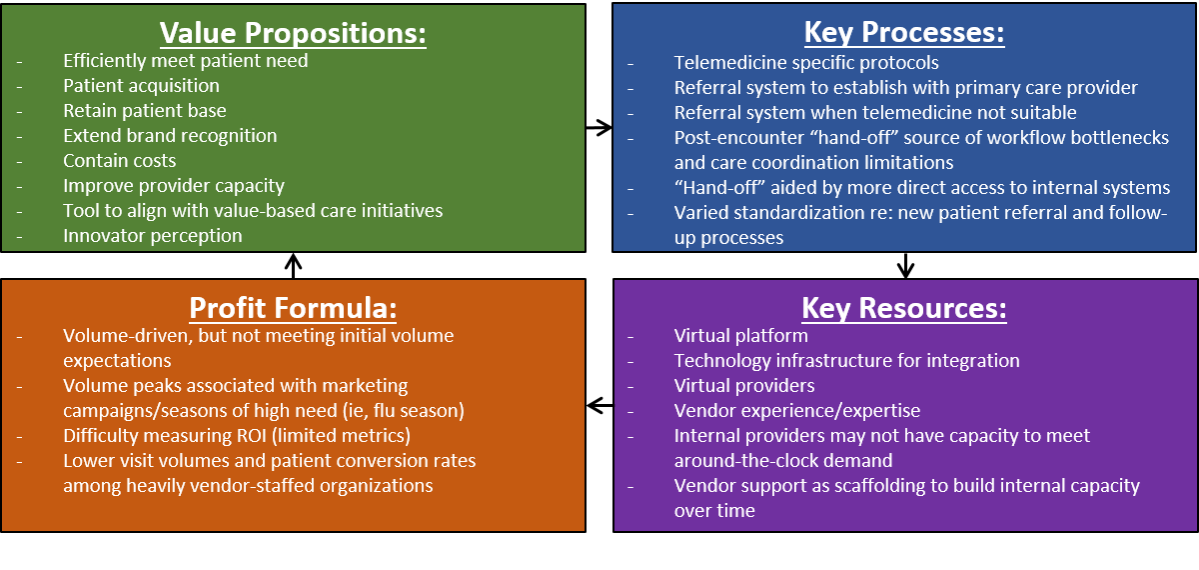
Summary of core strategic components of emerging business model archetype.

Figure 4 summarizes selected themes related to the 4 core strategic components (value proposition, key resources, key processes, and profit formula) that illuminate how VCC early adopter organizations are approaching the emerging business model we have described. We address each of these components below (a complete review of Figure 4 themes is included in Multimedia Appendix 3).

### Value Proposition

According to interviewees, a core leading value proposition for on-demand service launch was more efficiently meeting patient need to access care. For example:

The value proposition for us, really comes down to better service, easier access, faster access, being mobile, you know, being able to go right where those patients are, rather than having them come to us, and really the big keyword for all of [our goals] came down to access…

Other common value propositions included patient acquisition, retaining patient base, and extending brand recognition (often facilitated by white labeling of the VCC service by a telemedicine vendor). Regarding patient acquisition, an interviewee stated:

It's very expensive to acquire a new patient for health systems and so offering a convenient [virtual] urgent care and other consumer acquired services, it can be a very good way to acquire new patients and develop a new relationship with patients.

Reducing health care costs, or cost containment, also emerged as a frequent theme. One interviewee commented:

…there is an incentive for the health care system to be seeing patients in this way... I think it saves [the health system] money, it saves on unnecessary costs incurred by patients being seen when they didn’t have to be seen or … coming to an emergency room and utilizing resources that could better be utilized for patients who need that sort of in person service.

Some interviewees also identified improved provider capacity as a leading value proposition:

For us we are having a real access issue in our small rural county. And so we were using [VCC] as a way to provide services to our community whenever we don’t have provider capacity in our primary care clinic.

While less commonly expressed by interviewees, other value propositions also included the use of VCC as a tool to support population health management (in alignment with value-based care initiatives) or to promote an innovator perception to gain competitive advantage over peer organizations. Regarding promotion of an innovator perception, an interviewee commented:

…health systems see the value in extending their brand, and being seen as the leader in the market of telemedicine or virtual care, it allows them to differentiate in that manner…they see this as another arm in the overall machine of trying to generate new business for the organization.

### Key Resources

According to interviewees, common key resources among early adopter organizations include the VCC virtual platform, technology middleware to link clinical and administrative systems, and virtual clinical providers to staff the on-demand service. As reviewed in Table 2, many interviewees indicated that their organizations contract with third-party vendors to source some or all of these resources, particularly clinical staffing. Vendors were seen to provide vast experience and expertise to facilitate a fast and efficient VCC launch. For example, one interviewee explained:

I mean if it was just putting up a video chat component that’s not that difficult and anyone can do it but there is you know a lot of aspects to it, there's billing, there’s claims processing, there is integration to their systems, there is doctor availability, there is managing, training…so when you come to us you kind of get that complete package plus the expertise of you know what we have been able to accomplish over the past 10 years.

Many interviewees acknowledged that, for their organizations, pulling together key resources in-house requires extensive internal expertise regarding technology infrastructure and myriad aspects of virtual clinician staffing. While operational and clinical control was often identified as a perceived benefit, interviewees consistently indicated that it was challenging to meet around-the-clock patient demand for VCC with only their internal clinical providers. According to an interviewee:

Our intent is to staff it as much as possible with our employed providers. But it just doesn't make economic sense for us and we wouldn’t be able to maintain a low cost point if we're having to staff [the virtual clinic] at every low utilization time, for example in the early morning. And then also we wanted to be highly accessible not just in the states where…our patients are, but have it available to those patients as they travel out of state…so we have [a] partner network [with a vendor].

### Key Processes

According to interviewees, common processes among early adopters relate primarily to the VCC encounter, including use of telemedicine specific clinical protocols and systems for primary care referral and triage to in-person services. However, interviewees reported quite varied experiences in post VCC encounter processes. Among organizations relying primarily on clinically staffing support by vendors, interviewees described a patient hand-off process between the vendor, who provides the virtual encounter, and the adopter organization, who typically handles scheduling for new referrals and follow-up to check on patient progress after the clinical encounter. As one interviewee describes this hand-off process:

…I mean right now it’s a much more, I would say antiquated process, but the visit summary is sent to our [health information] department and then they are manually filing in that patient’s chart in the media tab…So that process of getting [a patient] set up with a primary care provider is outside of the [vendor] process.

According to interviewees, the lack of a standardized and strong hand-off process was associated with workflow bottlenecks and care coordination limitations:

Well ideally we would be able to get them in for a [visit] if they were hoping to have a primary care provider in our system. And so usually what ends up happening is we call them and get them on a wait list. It would be ideal if we could have more access and were able to actually pull them into our system.

Among those organizations that do not rely primarily on clinical staffing support by vendors, most interviewees reported that postencounter processes tend to be more standardized and efficient, greatly aided by more direct access to the internal systems of adopter organizations, especially electronic medical records (EMRs), referral systems, and appointment scheduling software. As an interviewee explains:

I think some health care systems are adopting this model and finding it better than hiring a [vendor] simply because having it done internally, people understand the internal process, they are already utilizing the same [electronic medical record] which ends up being a huge problem with hiring a [vendor] sometimes. And so the workflow and the integration and the follow up on patient care can be a lot easier when it’s done in-house rather than hiring one of these [vendors].

### Profit Formula

Overwhelmingly, interviewees described volume-driven profit generating mechanisms for VCC services, dependent on number of VCC encounters and referrals to other in-system services. However, with few exceptions, interviewees reported they are not meeting initial volume related goals:

I mean we’re satisfied with the quality and the customer satisfaction. We are not terribly satisfied with the volume for the growth trajectory…We thought it would grow faster than it did last year.

Volume peaks are commonly associated with VCC marketing campaigns and seasonal times of high need (ie, flu season). In general, interviewees representing organizations that rely heavily on vendor staffing typically reported lower encounter volumes and indicated less success at generating downstream volumes via patient conversion to primary care, compared with peers. As an interviewee explains:

[Patient] conversion is lower than what was targeted…I think we may have over projected potentially, initially on conversion.

### Review of Disruptive Business Strategies Employed by Early Adopters

Our qualitative study data revealed 4 business strategies that seem to particularly dictate the disruptive potential of VCC services, including the following: (1) fundamental disruptions to the model of care delivery; (2) outsourcing support for on-demand services; (3) disruptive market strategies to target potential users; and (4) new and unexpected organizational partnerships to increase return on investment.

### Fundamental Disruptions to the Model of Care Deliver: Modern Day Twist on House Calls

Interviewees’ comments regarding strategy focused on patient convenience, expediency, and appropriate level of care represent a fundamental disruption to standard models of care delivery. In fact, it can be viewed as a modern-day twist on the traditional house call. As an extension, to better facilitate the delivery of home care, many early adopters are incorporating home-based diagnostic testing and smartphone-based tools and peripheral devices to extend the capabilities and conveniences of VCC services:

I think we'll continue to see services evolve more and more to bring the online experience into a connected experience in the home…There are many devices available that you can attach to your Smartphone that would enable the provider to look in an ear or to listen to your heart or to listen to your lungs…and devices for home lab testing. So yeah it's something that we are keeping an eye on and then also thinking of how we can best utilize those to extend our services…[it’s] definitely something we are watching.

Regarding displacing traditional models, our data revealed a priority on right fitting care via the VCC care delivery model. One participating organization described placing VCC kiosks near emergency department waiting rooms to help triage patients to appropriate care settings based on medical need and patient choice:

We are looking at putting in a ER kiosk for virtual visits in one of our rural hospitals…that leadership team is wanting to have an option for those that really don’t need an ER visit that are using it more for primary care, to give them an option of a virtual visit…if it’s determined that really that patient does not need an ER visit, then they will be given options of seeing an ER physician, a same day appointment with the primary care doctor, urgent care option, or a virtual visit…and they’ll be given the cost.

### Outsourcing Support for On-Demand Services

As reported by many interviewees, early adopter organizations often outsource to third party vendors to launch, operate, and maintain their VCC services. According to our findings, outsourcing of clinical services is a relatively new and disruptive practice for adopting organizations. Early adopters reported varied and often flexible contracting relationships with vendors, particularly around support for clinical staffing. Although some limitations around the use of vendor services were noted, specifically lack of direct access to the internal EMR and billing systems of adopter organizations, vendor experience and expertise was largely considered a useful and agile resource for early adopters to expediently launch VCC services and to provide virtual clinical provider capacity for their VCC programs.

However, a complete dependency on external virtual clinical providers to staff the service line was not a permanent strategy. Many interviewees reported outsourcing strategies that utilized varying degrees of vendor support to provide important virtual provider scaffolding and increasingly bring the VCC service in-house as internal capacity improves and patient base expands. According to one interviewee:

While we could build it in house, our IT currently doesn’t have a skill set to be able to sport something of this magnitude…Now that being said, I know we are currently in discussions and are working on a plan, that hopefully within the next six to 12 months, that will start to combine [vendor] providers with our own.

### Disruptive Market Strategies to Target Potential Users

Owing to the patient-initiated nature of VCC and other on-demand telemedicine services, direct marketing to potential users emerged as a central and disruptive theme in the business strategies described by early adopters. Collectively, interviewees reported that VCC marketing strategies were largely new and uncharted terrain for their staff, distinct from the marketing needs for facility-based care delivery of in-person services:

Getting the name out there that was something we’ve never really had to do before. Because usually it’s just our name since health care is usually a new office, and [patients] already know what that health care is, [they] already know what an office does we don’t have to really educate or re-educate. [However, this was] a brand new product, brand new service, we had to get our name out there and educate [potential users] on what the product was and how it worked.

Interviewees overwhelmingly commented on the importance of *direct-to-consumer* marketing strategies to raise service awareness among potential users and ultimately drive service utilization and uptake. According to an interviewee:

We talk to clients about marketing all the time! Keeping that in their ear because, when it comes down to the bottom line, that’s what really drives utilization…Always, on our agenda every week we ask, what's your marketing, what discussions are you having, this did not work so what can we do differently to make sure it works.

Early adopters reported the use of varied marketing strategies, both traditional (eg, billboards and radio) and digital (eg, search engine optimization and websites). Interviewees reported marketing success when they prioritized funding and staffing for marketing efforts during initial VCC implementation as well as on an ongoing basis and utilized diverse marketing strategies, both traditional and digital. We further identified the value of marketing campaigns to specific seasons (eg, flu season) or opportunities of need (eg, part of information packets sent to new and relocated employees).

### New and Unexpected Organizational Partnerships

To increase opportunities for return on investment from VCC service launch, and to drive profit generation, many early adopters described new, and often surprising, partnerships with organizations outside of traditional health care delivery sector circles. For example, as discussed above, some interviewees commented on future plans to expand membership operations by partnering and contracting with self-insured organizations to offer VCC services directly and at a fee. According to an interviewee:

[Health systems are looking to] expand to a member program or a direct to employer program…there’s a huge opportunity there where a health system can go out and sell their brand name to these other organizations within the area.

As another example of the unique partnerships undertaken by early adopters, an interviewee discussed contracting with a nationwide hotel chain to offer VCC services to guests and employees. These new partnership strategies are innovative for the health care delivery sector and appear to be supporting many early adopters in their attempts to leverage value from their VCC services.

## Discussion

### Principal Findings

This qualitative study used the dominant instantiation of VCC to explore the paths that early adopter organizations are taking to harness the disruptive potential of on-demand telemedicine. In the coming years, this arguably disruptive form of telemedicine will seek to attract an early majority category of adopters. In turn, our findings contribute to the literature by providing insight for researchers and organizations considering launch or expansion of on-demand services to leverage what early adopter organizations have learned along the way regarding business model deployment. We also offer practical lessons learned regarding key strategy choices for adopter organizations as they launch on-demand services and encounter hurdles to value capture and delivery via deployed business models.

### Insights Into the Emerging Business Model for On-Demand Telemedicine

Health organizations have traditionally faced many struggles in aligning disruptive technologies with innovative business models [24-26]. To better understand whether organizations launching disruptive on-demand telemedicine services will meet a similar fate, this study explored emerging business models in the context of VCC early adopter organizations. With few exceptions, our study data suggest that current VCC early adopters are deploying value-adding process models that appear to appropriately match resources, processes, and profit formula to support value propositions for on-demand telemedicine.

By disentangling the reports from our interviewees regarding various business model archetypes, we were able to see a visionary progression of innovation among early adopters. Our findings demonstrate that business model archetypes and model components may evolve as organizations encounter challenges and opportunities related to VCC as a disruptive technology. In our study, we see many VCC early adopters that originally deployed a value-adding process model archetype beginning to transition to the use of a user-facilitated network model to better capture market share. To continue riding the wave of disruptive innovation and expansion spawned by on-demand telemedicine, early adopters are not staying stagnant: they are continuing to evolve their business models and recalibrate their core model components and strategies as new challenges and opportunities arise. Future research should pay particular attention to the deployment of user-facilitated networks, as many of the early adopters participating in our study indicated increasing use of this archetype as they explore new potential on-demand telemedicine innovations within their organizations.

### Strategic Direction: Strategy Helps to Transform the Business Model Into Action

We identified 4 strategy areas that seem to particularly dictate the disruptive potential of VCC services, including innovations in care delivery, outsourcing support, marketing strategies, and unique organizational partnerships. Below we review lessons learned for each of these strategy areas to help guide future practice for VCC and other forms of on-demand telemedicine.

#### Innovations in Care Delivery

Through much of the early 1900s, roughly half of all clinical visits involved a doctor coming into a patient’s home [43]. As health care systems grew larger, more specialized, and complex over the next century, the practice of the traditional house call became nearly nonexistent; facility-based, more expensive and often time-consuming models of care delivery, such as the physician office visit and emergency department, moved in to take its place [43]. On-demand telemedicine represents a fundamental change in the model of care delivery for patients—a modern-day re-envisioning of the traditional house call. Presently, VCC and other on-demand telemedicine services are pointing back to home care as a low-cost way to reduce time constraints, improve convenience and accessibility, and engage in shared decision making with patients to *right fit* care for common nonemergent conditions.

This new delivery model presents clear gains in convenience and accessibility for the treatment of many common, nonemergent medical conditions. However, when follow-up services are required to check on patient progress or to schedule patient appointments after the on-demand visit, our findings identified workflow bottlenecks and care coordination limitations within the postencounter process for many early adopter organizations. This may indicate a struggle to integrate home-based services into the larger continuum of care when patient contact and care coordination services are needed beyond the initial virtual visit.

There is limited guidance in the research literature regarding this integration process to inform decision making among adopting health organizations. However, lessons learned from our participating early adopters suggest that clinical integration of virtual visits into patient EMRs and other electronic systems to help track patient history and facilitate care coordination needs may be an important step to strengthen postencounter processes and the new care delivery model as a whole. Recently proposed policy by the Centers for Medicaid and Medicare Services—that will give patients access to their own downloadable health data [44]—may have implications that will break down barriers to the exchange of EMR data in the near future. The proposed initiative will potentially circumvent the EMR to empower health care consumers to share their health data with whomever they wish, including virtual providers.

#### Outsourcing Support

Among early adopters, outsourcing to third-party telemedicine vendors emerged as a key strategy to increase speed to market, gain access to technical infrastructure without taxing internal resources, and extend clinical staffing coverage for the on-demand service. Although interviewees described a variety of outsourcing contract arrangements, those that balanced internal resources with important scaffolding support from vendors appeared best suited to meet proposed value propositions. Outsourcing clinical services is still a relatively new concept to the health care delivery sector, and as such, there is limited guidance to inform future outsourcing decisions from telemedicine and health care sources. However, findings from the wider literature may prove instructive in the context of on-demand telemedicine [45-59]. Evidence-based guidance from the general outsourcing literature suggests adopter organizations should consider outsourcing a service in the context of low internal resources (particularly human resources) [48,49], the desire to increase flexibility regarding resources, operations, and other strategic elements [50], high internal costs (relative to expected costs of outsourcing) [51,52], and if other competitors are already outsourcing a given service [53]. In contrast, evidence suggests organizations should shy away from outsourcing a service in the context of high levels of market uncertainty [54], heavy integration of the service into internal systems [55,56], high level of service complexity [57], and if the service is considered a core competency to the service line [58,59]. We call VCC organizations and ensuing research to consider this evidence-based outsourcing guidance from other domains in exploring future strategies.

#### Marketing Strategies

Recent health care trends indicate overall telemedicine use is growing fast among patients but remains low overall [60]. These trends were echoed in what we heard from early adopters in our study, where most of the interviewees indicated that though their VCC service volumes were increasing, they were not meeting initial projections. Low utilization does not seem to be associated with usability issues [61,62] nor dissatisfaction [63], which have been identified as some of the more common barriers to technology adoption and use. In fact, many of our participants used patient satisfaction surveys as a means to measure satisfaction as an outcome and reported that patients that used VCC services were very satisfied. Upon investigating the few reports of dissatisfaction, the most often indicated underlying cause was the patient not receiving a prescription for antibiotics when they wanted one.

Instead, with few exceptions, early adopters connected their lower than expected VCC volumes to challenges around raising awareness for the service among potential users; to address awareness, interviewees often commented on the importance of *direct-to-consumer* marketing efforts. The importance of raising awareness of a new innovation is not new to disruptive technology research: awareness and knowledge generation is considered the first step in deciding whether to use a new innovation [14]. Not addressing awareness issues can impede adoption of consumer health technologies [64]. Increased awareness is often driven by the intersection of need recognition and marketing communications [14].

However, as was recognized in our study data, VCC marketing is largely new and uncharted terrain for early adopter organizations; according to an interviewee:

Getting the [VCC] name out there that was something we’ve never really had to do before. Because usually it’s just our [organization] name since health care is usually a new office, and [patients] already know what that health care is…

VCC marketing efforts seem to have a 3-fold purpose: (1) to provide the health consumer with understanding about the availability of VCC; (2) educate the health consumer about the medical situations when VCC is a good option; and (3) *sell* the health organization as this is where a strong link needs to be created for the health consumer to turn to the health system’s VCC offering among other options. Regarding education, as with some other early innovations (eg, LinkedIn), potential adopters may not understand all of the uses and potential of VCC.

Marketing in the form of health system *branding* is still relatively new, and marketing direct-to-consumer services like VCC are even newer. In cases of one-time or episodic care similar to VCC (where the patient may not always interact with the same provider), research suggests that the presence of tight bonds between patients and a sponsoring organization, or even organizational representatives, is a key facilitating factor for successful telemedicine service interactions [61]. This finding has important potential implications for organizations as they market their VCC services. First, organizations should consider directing their marketing efforts not only toward potential virtual patients but also organizational representatives (ie, primary care providers, other staff) who may share their existing close bonds with their patients and can function as pseudo brand ambassadors to raise awareness of VCC services. We also learned in our conversations with interviewees of some limited activity in this area, particularly in regard to adopter organizations asking physicians to post VCC advertisements in their offices. Second, it indicates that as health organizations continue to expand and strengthening their *health organization branding*, they should leverage their organizational brand in their marketing efforts to raise awareness for VCC; they should consider marketing VCC not as a separate product, but instead as an available service offered by an organization that patients already know and trust to manage their medical care. Building this type of patient-organization connection is still relatively new and evolving, as patients are generally more welded to individual providers rather than to health organizations. Adopting provider organizations, such as health systems, may have an advantage in leveraging patient-organization relationships to raise VCC awareness because of their potential role as a regular source of in-person care for patients and as a well-known health care institution in local communities. We see that some early adopters are already engaging in this activity by working with vendors to white-label their VCC services so that they may present the service with strong health system branding.

However, early adopter organizations should also recognize important externals factors that may present challenges to ongoing marketing efforts to raise VCC awareness and drive utilization; namely, limited telemedicine reimbursement that may prevent penetration to certain patient markets (eg, Medicare patients), and provider credentialing and other regulations that may prevent organizations from providing services across state lines [65,66]. Although recent policy changes have reduced these limitations [67], policy barriers are not completely eliminated, and those still challenge the capabilities of health organizations adopting VCC to expand virtual service offerings and grow their patient volume.

Although, in our study, we identified a number of strategies that led to greater marketing success among VCC early adopters to drive uptake (eg, using both traditional and digital strategies), there is little additional evidence-based guidance to inform future strategic decision making in the health care marketing literature, creating an opportunity for future work. Future research efforts may be informed by research exploring factors to help organizations design, manage, and market service delivery interactions for medical video conferencing, a different form of telemedicine [68].

#### Unique Partnerships

According to interviewees, early adopter organizations are particularly motivated to explore innovative relationships with external entities to increase the opportunity for return on investment and profit generation related to on-demand telemedicine services. Reviewed above, a prominent example of this involves early adopter health systems contracting with self-insured organizations to offer VCC services directly. Examining other emerging and unexpected partnerships between health care and business entities, such as the recent formation of a health care company between Amazon, Berkshire Hathaway, and JPMorgan, may help to shed some light on how these innovative organizational relationships will influence the direction of VCC and other telemedicine services in the future. With the goal of improving health care services and cutting costs for more than 1.1 million employees, the Amazon partnership is predicted to disrupt the health care marketplace by using technology solutions to develop innovative treatments and modernize delivery system processes [69]. Similarly, new partnership arrangements related to VCC and other on-demand telemedicine solutions also have the potential to disrupt health care. It remains to be seen how these new organizational relationships may impact the use of various business model archetypes and strategies for new technologies in health care.

### Study Limitations

Our focus on a narrow study population of VCC early adopter organizations may limit the generalizability of our study findings. As a result, some findings may not be applicable to other forms of on-demand telemedicine, such as behavioral health. In addition, we did not study nonadopters or organizations with failed VCC adoption experiences; learning about the experiences and challenges faced by these organizations would have provided additionally meaningful insights to address our research objective. Our use of a convenience and purposive sampling approach may also present limitations to study generalizability. Although we targeted organizations in different geographic areas and of varying size and type, it is possible that the perspectives of some VCC early adopters are not represented in our study dataset. It is also possible that given time constraints, lack of knowledge, or hesitancy to discuss business information, key informants may not have shared some details of potential interest to researchers. However, key informants were generally very open and forthcoming during study interviews, thus reducing concerns that important themes may not have been revealed. They were eager to share and indicated they were motivated to learn from our findings as a means to further advance their VCC program efforts. Finally, we specifically targeted *early adopters*, representing only a minority of potential adopters along Rogers diffusion of innovation curve [14]. However, the purpose of our study was to offer guidance to new organization entrants as they consider viable business models and strategies for on-demand telemedicine, necessitating an exclusive focus on early adopters.

### Conclusions

Current trends suggest health organizations will increasingly use on-demand telemedicine as a means to meet patient demand for convenient, accessible, and affordable services, and to address other leading health care challenges. Here we presented on-demand telemedicine as a potentially disruptive innovation in the early adopter stage of technology adoption and diffusion. For the research community, we contributed a new level of contextualization to disruptive innovation research targeted to the health information technology space. For early adopters, the insights we have shared can help organizations navigate evolving opportunities and address challenges to leverage their position of early entry. However, to truly be a positive disruption that will increase accessibility and affordability for health care consumers, on-demand telemedicine must cross into the early majority stage of widespread assimilation. For potential early majority organizations that are considering launch of on-demand services, insights from this study provide an opportunity to leverage what early adopters have already learned along the way to mitigate unknowns and risks as they deploy innovative business models and make strategy choices to harness the disruptive potential of on-demand telemedicine.

## Appendix

Multimedia Appendix 1 Overview of virtual urgent care clinic patient encounter process.

Multimedia Appendix 2 General interview protocol.

Multimedia Appendix 3 Summary of core strategic components of emerging business model archetype.

## Abbreviations

EMR: electronic medical record
VCC: virtual urgent care clinic
