# Supplementary material for: On-Demand Telemedicine as a Disruptive Health Technology: Qualitative Study Exploring Emerging Business Models and Strategies Among Early Adopter Organizations in the United States
Source: J Med Internet Res. 2019 Nov 15;21(11):e14304. doi: 10.2196/14304 (PMC6884714; doi:10.2196/14304)
Supplement: Multimedia Appendix 1 [file jmir_v21i11e14304_app1.docx]

#### Appendix 1: Overview of VCC patient encounter process

In the typical VCC encounter, a patient in need initiates a request for a virtual visit using a web browser, phone, or tablet. The patient will complete an online intake form, detailing medical history, presenting symptoms, and primary care provider (PCP) status; if no established PCP is indicated, the patient is prompted to indicate if they would like a referral request. After completing intake, a patient will typically connect with a virtual provider within 10-15 minutes. Based on chief medical complaint and history, the provider will treat the patient through the virtual visit (may include prescribing medications) or, if clinically indicated, triage the patient to a form of in-person care for immediate consultation or recommended follow-up.
