# Supplementary material for: On-Demand Telemedicine as a Disruptive Health Technology: Qualitative Study Exploring Emerging Business Models and Strategies Among Early Adopter Organizations in the United States
Source: J Med Internet Res. 2019 Nov 15;21(11):e14304. doi: 10.2196/14304 (PMC6884714; doi:10.2196/14304)
Supplement: Multimedia Appendix 2 [file jmir_v21i11e14304_app2.docx]

#### Appendix 2: Interview protocol

**Virtual Care Clinic Interview Prototype (General Interviews)**

**Length of Interview**: 45-60 minutes **Milieu**: Telephone

**Methodology**: An email interview request with an overview will be sent to participants in advance of the interview to allow for proper preparation and effective use of interview time. Interviews will be recorded and notes will be taken. The interview is discreet. Results will be de-identified and only the interviewers will have access to the transcripts.

Dial-in Number:  712-770-4700
Access Code: 259459

Host PIN code: 6856

Note that the questions listed are a bank of questions desired for each participating organization.

Key questions to be asked of all interviewees.

Prompts -- Prompts are supporting questions and are listed under the main questions (indented). Prompts are asked if the respondent does not seem to understand the main question address the main question as expected.

**Introductions to INTERVIEWERS**

- Thank you for setting aside time for this interview.
- ________ (interviewee name), it is a pleasure to speak with you.
- We can start with introductions
- My name is _______ (Cynthia or study research assistants). I am a _____ (Cynthia or study research assistants) with the University of Washington and am currently working with the National Science Foundation’s Center for Health Care Transformation to identify best practices and lessons learned from early adopter organizations of virtual care clinics. My role is to serve as an independent expert to conduct interviews and analyze the resulting information.
- I would like to also introduce ________ (Cynthia or study research assistants), from my team, who is present to listen, take notes, and interject follow-up questions at appointed times, in the event clarification is needed.
- Would you like to introduce yourself?

**Introduction to the Interview (adapt to who speaking with and prior COMMUNICATION and materials sent):**

- The focus of this interview is to understand your experiences and lessons learned. We are speaking with representatives of health organizations across the country that offer virtual care services so that we can identify important insights to share around implementation, operations, and patient engagement.

**The process**

- The interviews will be completely confidential. Your identity will only be known to interviewers on the research team. You or anyone you refer to during this interview will not be mentioned by name nor will your site be identified by name. Interview results will be de-identified before they are analyzed or reported in order to protect confidentiality. Your participation in this research is completely voluntary. You may decide to stop participating in the research at any time. Do you have any questions regarding the interview process or the treatment the information that will be collected at this time?
- ______ (Cynthia or study research assistants) will serve as the lead interviewer.
- We have grouped these questions into categories to facilitate the flow of the conversation.
- ________ (Cynthia or study research assistants) will break for a moment at the end of each category to see if _____ (Cynthia or study research assistants) has any follow-up questions or would like to clarify any comments made.
- We would like to audio record the interview with your permission to ensure that we capture your thoughts accurately and allow us to fully focus on the conversation with you, rather than on note taking. If there are any comments that you do not want to have recorded, just let us know and we can postpone discussion until the recorder is turned off. Do you have any problems with recording? We will now turn on a recorder to capture the interview.

**TURN ON THE RECORDER**

- I am now recording with your permission.

**OVERVIEW OF ORGANIZATONAL ROLE (announce this and forthcoming categories to interviewee):** I’d like to start by learning a bit more about you and your organization.

1. What is your role within ___________ (organization)? How does telemedicine in general fit with your role?
   1. What about virtual care services?

**STRATEGY AND VISION:** These next questions are related to the scope and background of VCC services at your organization.

1. What motivated VCC launch within your organization?
   1. Was the decision influenced by your organization’s strategic plan?
      1. Related to cost containment efforts?
      2. Related to patient care?
      3. Related to patient acquisition?
   2. Was the decision influenced by the nature of your organizational culture?
      1. Embrace new ideas?
      2. Be a leader in innovation?
   3. Was the decision related to the desire to expand existing services?
      1. Size/maturity?
      2. Preexisting experience/knowledge?
   4. Was the decision related to the desire to compete with peer organizations or other service options?
   5. Did any state or organizational policy change influence the decision?
      1. Reimbursement changes?
2. Tell me about VCC services at your organization?
   1. How long has your organization offered VCC services?
   2. What is the extent of current VCC service provisions?
   3. Has this changed since initial VCC launch?
3. How are VCCs services funded within your organization?
   1. Are VCC services funded through a core budget?
   2. Funded through a grant initiative?
   3. Funded as a pilot program?
4. How can patients access VCC services?
   1. Is this something you contract out to a vendor company?
   2. What telecommunication modalities can patients use?
   3. Are patients referred to follow-up care services if needed??
   4. Is a care summary sent back to a patient’s primary care provider (if the information is available)?

**PATIENT ENGAGEMENT:** Now we’re going to transition to a set of questions that focus on the patient side of VCC services at your organization.

1. How are patients made aware of VCC services?
2. How are you currently engaging with patients to encourage VCC service use?
   1. Do you (or have you) made any formal marketing efforts?
      1. Do you target specific patient groups through those efforts? Let’s talk about that.
      2. Is there any document or marketing materials you would be willing to share with us?
   2. Or are engagement efforts more passive?
   3. Have efforts proven successful?
   4. How do you track patient engagement?
   5. What would you say are important barriers to engaging patients related to their use of VCCs services?
   6. What about important facilitators?
3. What has patient uptake been like for VCC services?
   1. What has patient uptake looked like over time? Are you satisfied with where uptake is at this point?
   2. How would you describe a typical user of VCC services?
   3. What are some of the most frequent reasons patient’s use these services?
   4. What are some of the barriers you’ve had to overcome to get patient uptake to where it is today? What remaining barriers do you have to overcome to get uptake to where you ultimately want it?
      1. How have you overcome these barriers (or how do you think you can overcome these barriers)?
   5. What are some of the reporting mechanisms you use to collect and track patient uptake?
4. How do patients that use VCC services respond to the service?
   1. Would you say current services are meeting patient needs?
   2. Have patients reported any difficulty accessing and/or using services?
      1. Related to technology?
      2. Related to interactions with medical providers?
      3. Related to the operational process?
   3. How would you say the VCC experience at your organization compares to similar services offered through other access points? For example, similar services might include face-to-face primary care, or services received at urgent care centers or convenient care clinics.
   4. What are some of the reporting mechanisms you use to collect and track patient experience information?

**IMPLEMENTATION PROCESS:** Now we’re going to focus specifically on how VCC was implemented at your organization.

1. Who supports VCC implementation and operations?
   1. Is everything outsourced? (if vendor company is used)
   2. Is there a department or service line that supports these activities?
      1. What does the staffing look like associated with the department or service line?
   3. How are decisions made related to VCC implementation and operations? How is information exchanged between stakeholders?
      1. Vertically?
      2. Workgroup structure?
   4. What are some of the reporting mechanisms you use to collect and track information related to the impact of VCC implementation and operations?
2. In looking back on the implementation process, what were some things that were instrumental?
   1. Leadership?
   2. Champions?
   3. Technical support?
   4. Funding?
   5. What would you say got in the way of successful implementation?
3. If you were going to advise someone just starting this program, what would you tell them based on what you’ve learned to date?

**Closing reflections:** We’re just about done. Now we’re going to wrap up with a few big picture questions related to lessons learned and the future of VCC services at your organization.

1. What suggestions do you have for current and future VCCs within other health organizations based on what you have learned to date?
2. So what does the future look like for VCC within your organization? What are the critical success factors moving forward?
3. Are you using VCC services to leverage any other opportunities?
   1. Expanded telemedicine services?
   2. Community benefit?
   3. Grants?
   4. Patient acquisition?
4. Are there topics or issues regarding VCC implementation, operations, and patient engagement that we have not covered would be important to understand?
5. Is there anyone else that you would suggest that it would be important to speak with to learn more about VCC services at your organization?

**Conclusion**

- That concludes this interview.
- Thank you for taking the time out of your busy schedule to meet with us. Speaking with you was both enjoyable and informative.
- In the remote chance that I have a follow-up question to gain clarity after reviewing the notes or transcripts, would it be ok to reach out to you via email?
- Also, as the project continues, would it be ok to check back with you regarding updates and additional insights?

**TURN OFF THE RECORDER.**

- IMMEDIATELY download the recorded file.
- Once the file has been checked, remove the recorded file from the pass key web site.
- Send recording to transcription service.
