# Supplementary material for: On-Demand Telemedicine as a Disruptive Health Technology: Qualitative Study Exploring Emerging Business Models and Strategies Among Early Adopter Organizations in the United States
Source: J Med Internet Res. 2019 Nov 15;21(11):e14304. doi: 10.2196/14304 (PMC6884714; doi:10.2196/14304)
Supplement: Multimedia Appendix 3 [file jmir_v21i11e14304_app3.docx]

#### Appendix 3. Summary of core strategic components of emerging business model archetype

| **Core strategic component** | **Supporting quotes** |
| --- | --- |
| Value proposition | **Efficiently meet patient need:**  “The value proposition for us, really comes down to better service, easier access, faster access, being mobile, you know, being able to go right where those patients are, rather than having them come to us, and really the big keyword for all of [our goals] came down to access…”  “…so [telemedicine] has more so become an additional door or an access point rather than this you know shinny object that lets you do video chat and so in terms of the value I think they are trying to figure out how to be more efficient and they are using virtual care in many ways to support that goal.”  **Patient acquisition:**  “It's very expensive to acquire a new patient for health systems and so offering a convenient [virtual] urgent care and other consumer acquired services, it can be a very good way to acquire new patients and develop a new relationship with patients.”  **Retain patient base:**  “A lot of times when they have a risk contract and they're responsible for the life of the patient, they're also interested in retaining patients, so they'd rather not see that patient go off to another organization and get even urgent care from the other organization because that very encounter can lead to, "Okay, actually you need to be seen. Why don’t you come in to our other facility…" and then care becomes discontinuous for the health system. So that, I think is a really important value driver.”  **Extend brand recognition:**  “They are looking to be a brand in the market, they are looking to expand beyond their four walls. They are trying to cast a larger net so to speak.”  **Contain costs:**  “…there is an incentive for the health care system to be seeing patients in this way... I think it saves them money, it saves on unnecessary costs incurred by patients being seen when they didn’t have to be seen or … coming to an emergency room and utilizing resources that could better be utilized for patients who need that sort of in person service.”  **Improve provider capacity:**  “For us we are having a real access issue in our small rural county. And so we were using [VCC] as a way to provide services to our community whenever we don’t have capacity into our primary care clinic.”  **Tool to align with value-based care initiatives:**  “…it’s really more about the move from a pay for service model of care to a value model. And in that value model when doctors aren’t paid anymore on RVU’s and aren’t paid for the procedures that they do and it’s not about volume they are not going to care to necessarily see the three year old with pink eye because it ends up being a waste of their time. And we need to free them up so that they are dealing with the more complex cases. And that’s a much longer road to get there and has a lot more… everybody has to move to value because the model of fee for services is broken”  “Now long-term as healthcare is trying to move to value where it becomes more and more important to really offer a cost efficient and high quality product to existing patients and especially to the rich population. Our strategy will be augmented and extended in that sense to offering to the existing and risk population. But right now at this point it's really a new patient acquisition tool.”  **Innovator perception:**  “…health systems see the value in extending their brand, and being seen as the leader in the market of telemedicine or virtual care, it allows them to differentiate in that manner… they see this as another arm in the overall machine of trying to generate new business for the organization.” |
| Key resources | **Virtual platform:**  “They can come through phone or if they want to self-select on the website they can do so in that way. It’s a brief in-take of 4 or 5 pages, that they list out how they are feeling, how they would like to receive care, they select their primary care provider if they have one in the health system, then consent and okay, and depending on what modality they choose they would enter an exam room for an outbound call from a provider or a video call.”  **Technology infrastructure for integration:**  “…so I think most important thing is the integration of the EMR…that to me in terms of being a clinician, having the continuity of care, having the record in place, being able to access medical history and medication and things like that in any EMR integration of data… it makes a difference in what we do and how we provide that care.”  **Virtual providers:**  “I’m thinking of the providers that I’m bringing on, what truly makes them really good, it’s that passion for care, I don’t think there is anything different than any other providers out there in any clinic settings if you will…Using the technology is another avenue to bring care to the patient.”  **Vendor experience/expertise:**  “I mean if it was just putting up a video chat component that’s not that difficult and anyone can do it but there is you know a lot of aspects to it, there's billing, there’s claims processing, there is integration to their systems, there is doctor availability, there is managing, training…so when you come to us you kind of get that complete package plus the expertise of you know what we have been able to accomplish over the past 10 years.”  “The reason that we went with a vendor was speed to market. In order to build [the platform] ourselves it would have taken longer and we wanted to get out in the market place as quickly as possible.  **Challenge to meet around-the-clock demand using internal providers:**  “Our intent is to staff it as much as possible with our employed providers. But it just doesn't make economic sense for us and we wouldn’t be able to maintain a low cost point if we're having to staff [the virtual clinic] at every low utilization time, for example in the early morning. And then also we wanted to be highly accessible not just in the states where … our patients are, but have it available to those patients as they travel out of state … so we have [a] partner network [with a vendor].”  **Vendor support as scaffolding to build internal capacity over time:**  “While we could build it in house, our IT currently doesn’t have a skill set to be able to sport something of this magnitude, plus sometimes you know, in the time it takes us to learn, and hire, and train, and figure out all the best practices and all that and the other thing, and get this all up and running and smooth, you know, that’s a big piece. Sometimes you got to just rely on a company that’s just done it many times before, and have other clients and have the technical support at your fingertips… Now that being said, I know we are currently in discussions and are working on a plan, that hopefully within the next six to 12 months, that will start to combine [vendor] providers with our own” |
| Key processes | **Telemedicine specific protocols:**  “There is a pretty standard protocol in terms of caring for the patient during the actual examination itself.”  **Referral system to establish with primary care provider:**  “…if [the patient] had told us during the registration process that they wanted a PCP they would be immediately warm transferred to our centralized scheduling [team].If they told during the registration process that they didn’t have a PCP but they didn’t want one then we get a report within four hours that says who those patients are that don't have a PCP and we and we reach out to them and we talk to them about the benefits of a primary care provider.”.  **Referral system when telemedicine not suitable:**  “Obviously we see some conditions that are not virtually appropriate or cannot be managed virtually so that would warrant a referral back into the health system.”  **Post-encounter “hand-off” source of workflow bottlenecks and care coordination limitations:**  “Well ideally we would be able to get them in for a [visit] if they were hoping to have a primary care provider in our system. And so usually what ends up happening is we call them and get them on a wait list. It would be ideal if we could have more access and were able to actually pull them into our system.”  **“Hand-off” aided by more direct access to internal systems (e.g., EMR):**  “I think some health care systems are adopting this model and finding it better than hiring a [vendor] simply because having it done internally, people understand the internal process, they are already utilizing the same [electronic medical record] which ends up being a huge problem with hiring a [vendor] sometimes. And so the workflow and the integration and the follow up on patient care can be a lot easier when it’s done in-house rather than hiring one of these [vendors].”  **Varied standardization re: new patient referral and follow-up processes:**  “Clearly where we have an integration with the EHR then we're able to instantiate a new patient into the health record for the health system. But the health system may have a process where they want us to refer the patient to a referral number by phone. They may have a process where they're going call back the patient, then they have a process whereby we do a warm transfer. It's... there are a variety of processes that happen”  “We do follow phone calls; oh that's a lot of differentiation. We actually call the patient. We call every patient to follow up with them and make sure that what we… what we prescribed is actually making a difference for them.” |
| Profit formula | **Volume-driven, but not meeting initial volume expectations:**  “I mean we’re satisfied with the quality and the customer satisfaction. We are not terribly satisfied with the volume for the growth trajectory …. We thought it would grow faster than it did last year.”  “[Patient] conversion is lower than what was targeted… I think we may have over projected potentially, initially on conversion. “  **Volume peaks associated with marketing campaigns/seasons of high need:**  “And in one of my favorite campaigns we did, we did at Christmas…and we set up a booth and we had our door price and our big sign that had Santa on it and was pretty much who does Santa call when he gets sick? Well, we all know who Santa is? So it was a big draw…”  **Difficulty measuring ROI:**  “So a lot of hospitals systems that we meet with us say, ‘Well we did the math and it cost X to use your technology and what we found is we don’t believe we'll be able to do more than X number of visits.’ So they tie a lot of it into how many visits they're going to do and what that exact revenue is. That's not the way we've been looking at it because as I mentioned it’s still a nascent space and a lot of this is arbitrary. So I think that when we get a health system that’s being realistic and thinking about this in a big picture way, I think our measure of success is having a solution that's you know branded to them and works really well.” |
